# Behavioral Interventions for Children and Adolescents With Fetal Alcohol Spectrum Disorders

**Published:** 2011

**Authors:** Blair Paley, Mary J. O’Connor

**Keywords:** Prenatal alcohol exposure, fetal alcohol spectrum disorders, fetal development, developmental disability, developmental disorder, teratogenesis, child, adolescent, intervention, behavioral intervention

## Abstract

Exposure to alcohol in utero is considered to be a leading cause of developmental disabilities of known causation. The most severe consequence of such exposure, fetal alcohol syndrome (FAS), is characterized by a distinct constellation of facial anomalies, growth retardation, and central nervous system dysfunction. Both animal and human studies, however, suggest that there may be considerable variability in the manifestations of in utero alcohol exposure across individuals, and, consequently, the term fetal alcohol spectrum disorders (FASD) has come into usage to reflect the entire continuum of effects associated with such exposure. In addition to FAS, this term encompasses the conditions of partial FAS, alcohol-related neurodevelopmental disorder, and alcohol-related birth defects. Despite extensive evidence of significant cognitive, behavioral, and social deficits in people with FASD, research on behavioral interventions for FASD has lagged behind. However, in recent years there has been a marked increase in efforts to design and test interventions for this population. This article will review current empirically tested interventions, methodological challenges, and suggestions for future directions in research on the treatment of FASD.

More than 35 years ago, fetal alcohol syndrome (FAS) was first identified in the United States as a major birth defect resulting from prenatal alcohol exposure (Jones and Smith 1973; Jones et al. 1973). FAS is characterized by a distinct constellation of characteristic facial anomalies, growth retardation, and central nervous system dysfunction. Evidence from both animal and human studies, however, suggests that there is considerable variability in the manifestations of in utero alcohol exposure across individuals. Such variability depends on numerous factors, including dosage, timing of exposure, pattern of exposure, maternal age and body mass index and genetics, as well as postnatal variables such as nutrition, socioeconomic conditions, and environmental enrichment (Bonthius and West 1990; Day and Richardson 2004; Downing et al. 2009; Hannigan et al. 2007; Jacobson et al. 2006; Jones 2006; May et al. 2008, see also May and Gossage, pp. 15–26, in this issue). In light of this variability, the umbrella term, fetal alcohol spectrum disorders (FASD) (Warren et al. 2004) has come into usage to reflect the entire continuum of effects associated with in utero alcohol exposure. In addition to FAS, this term encompasses the conditions of partial FAS, alcohol-related neurodevelopmental disorder (ARND), and alcohol-related birth defects (ARBDs, as described by the Institute of Medicine [Stratton et al. 1996]).

Over the past three decades, extensive research has documented the teratogenic effects of alcohol in both animal and human studies, and such research has highlighted a range of cognitive, behavioral, and physical impairments associated with prenatal alcohol exposure. Intellectual and learning disabilities, executive dysfunction, speech and language delays, behavioral and emotional difficulties, poor social skills, and motor deficits have all been reported among people with FASD (Burd et al. 2003; Green et al. 2009; Guerri et al. 2009; Kalberg et al. 2006; Kodituwakku 2007, 2009; O’Connor and Paley 2009; Paley and O’Connor 2007; Rasmussen 2005; Rasmussen and Bisanz 2009; Riley and McGee 2005; Roebuck et al. 1998; Streissguth 2007; Streissguth et al. 2004; Walthall et al. 2008; Willoughby et al. 2008).

Notably, much of the FASD research has focused on people receiving treatment, often at clinics specializing in FASD diagnosis and treatment. Therefore, less is known about how people exposed to alcohol prenatally might present in more generalized mental health or medical settings, where their impairments may be less readily identified as resulting (at least partly) from in utero exposure to alcohol. More population-based studies are needed to identify alcohol-exposed individuals who exhibit significant impairments but cannot access medical or mental health services, as well as those who exhibit milder (or more subtle) effects of such exposure. Recent studies focusing on school-based samples in the United States, Europe, and South Africa have found that children with FASD perform significantly worse on measures of cognitive and adaptive functioning when compared with children without FASD (Adnams et al. 2001; Aragon et al. 2008; Kodituwakku et al. 2006*a*, *b*; May et al. 2009). Such findings offer preliminary evidence that the impairments seen in children with FASD are not limited to those seen in clinical settings, but additional epidemiological studies would help clarify the full range of outcomes for individuals with a history of in utero alcohol exposure.

From economic, societal, and family perspectives, FASD represent a major public health issue. Prevalence estimates vary depending on the method of ascertainment and the populations sampled. A recent review by May and colleagues (2009) estimates the prevalence of FAS in the United States to be at least 2 to 7 per 1,000 in typical, mixed-race populations of mixed socioeconomic status. Prevalence estimates for the entire continuum of FASD range from 1 to 5 percent (May et al. 2009; Sampson et al. 1997), with similar rates documented in other Westernized countries (Elliott et al. 2008). Even higher rates have been documented in countries with high rates of poverty, such as South Africa (May et al. 2007; Viljoen et al. 2005). Taken together, these findings suggest that these conditions are more common than previously thought and, notably, more common than a number of other more well-known developmental disabilities. Previous estimates regarding the annual cost of FAS also have varied, ranging anywhere from $75 million to $4 billion per year, with such wide variations likely a function of several factors, including what types of costs are included, the age range covered, and prevalence rates in the population examined (Lupton et al. 2004). In the United States, the lifetime cost for an individual with FAS has been estimated to be approximately $2 million, the majority of which reflects costs for special education and medical and mental health treatment (Lupton et al. 2004). Research in other countries has documented similarly high costs of these conditions (Stade et al. 2009; Thanh and Jonsson 2009). Although the costs for all conditions on the FASD continuum are unknown, they are expected to be considerably higher (Lupton et al. 2004). People with FASD are at a greatly increased risk for a host of secondary disabilities, including school failure, delinquency, and alcohol and substance abuse problems (Alati et al. 2008; Streissguth et al. 2004). Families of individuals with FASD often are faced with a scarcity of diagnostic and treatment services, professionals who do not fully comprehend the nature of their children’s disability, and inadequate social support (Olson et al. 2009).

Growing recognition of the widespread and often multigenerational impact of FASD has fortunately served as a major impetus for the development of behavioral interventions that aim to address both primary deficits and secondary disabilities in this population and ultimately improve the quality of life for these individuals and their families. This article will discuss challenges in developing and testing such approaches for people with FASD and their families, review recent studies examining the efficacy of a variety of behavioral approaches, and highlight potentially fruitful directions for future research on the treatment of FASD.

## Challenges in Developing Effective FASD Interventions

Researchers and clinicians working with the FASD population have highlighted the importance of recognizing that the impairments often reported in people with FASD may be a function of multiple contributing factors (Coggins et al. 2007). Other prenatal risk factors may include exposure to other teratogens, poor prenatal care, poor maternal nutrition, or maternal stress during pregnancy. Moreover, these individuals also may experience postnatal environmental risk factors, including ongoing parental substance use/abuse, parental psychopathology, exposure to interparental conflict or domestic violence, and neglect or abuse. Several studies have shown that many children with FASD experience one or more changes in custody during their lives, such as being placed in foster care or put up for adoption or being institutionalized (see Stratton et al. 1996). Not surprisingly, children with FASD are overrepresented in foster care populations. Astley and colleagues (2002) found the rate of FAS to be 10 to 15 times higher for children in foster care than in the general population. Although being removed from the biological home in which a parent still is abusing alcohol may reduce some risks for these children, they nonetheless remain vulnerable to adverse environmental experiences, including disruptions in caregiving relationships. Moreover, in utero alcohol exposure may operate in concert with other adverse circumstances to confer further risk on these individuals. For example, a recent study (Smith et al. 2007) found that a history of prenatal alcohol exposure increased children’s risk for a higher number of foster care placements and for maltreatment. Placement instability likely plays a major role in further exacerbating the medical and mental health difficulties of these children and reducing access to adequate services by causing disruptions in caregivers and health care providers (Mekonnen et al. 2009).

In light of the multiple risks often experienced by children with FASD, designing effective interventions may be challenging. Interventions that seek to both remediate primary deficits as well as mitigate the various environmental liabilities that often accompany a history of prenatal alcohol exposure may yield the most positive outcomes. Such approaches may necessitate focusing not only on the child but on their caregiving environment as well. A number of intervention approaches described in this article have included efforts to improve parenting skills, train parents and caregivers to better advocate for services and connect with community resources, and/or directly enhance caregiver functioning. Other approaches have involved teaching parents strategies to promote and reinforce targeted skills at home and in other settings in order to promote generalization and maintenance of treatment gains.

Identifying people who have been affected by in utero exposure to alcohol presents another challenge to developing and implementing effective FASD interventions. Part of this challenge relates to a continuing need to improve FASD training among health care providers. Some providers may lack training in how to ask patients about prenatal alcohol exposure, in recognizing the features of FASD, and in making a diagnosis or knowing where to refer a patient for diagnosis (Elliott et al. 2006; FASD Regional Training Centers Consortium 2007; Gahagan et al. 2006; Paley et al. 2009). Stigmatization associated with FASD also may create obstacles in obtaining an accurate history of a child’s exposure to alcohol prenatally. Practitioners may be uncomfortable asking about prenatal alcohol use or not well trained in the most effective strategies for asking about such history. Mothers may be disinclined to fully disclose their history of alcohol use during pregnancy because of concerns that their health care provider will respond in a blaming or judgmental fashion, or they may be worried that there could be potential legal repercussions of such disclosures.

As a result of such obstacles, many people with FASD are not referred for diagnosis until relatively late (if ever) and thus miss out on the potential benefit of early intervention. For example, findings from the Washington State FAS Diagnostic and Prevention Network revealed that the average age of referral for diagnosis in their clinical sample was 9.5 years (Olson et al. 2007). Although the age at which individuals are identified or diagnosed likely varies from setting to setting, there remain significant concerns that FASD are underrecognized and undertreated, particularly in certain high-risk settings, including psychiatric hospitals, the child welfare system, and juvenile detention and correctional facilities (Astley et al. 2002; Burd et al. 2004; O’Connor et al. 2006*a*). By the time many of these individuals are identified and receive treatment, a pattern of significant behavioral and emotional problems, poor school functioning, and negative family interactions already may be well established. The importance of early identification is highlighted by findings from Streissguth and colleagues (2004) that an earlier diagnosis is one of the strongest predictors of more positive outcomes for alcohol-exposed individuals.

## Behavioral Interventions for People With FASD

Despite the recognition of FAS in the United States for more than three decades (Jones and Smith 1973) and the wealth of studies documenting the teratogenic effects of alcohol on multiple domains of functioning, research-based interventions for this population are lacking (Burd 2006; Premji et al. 2006). Recently, however, both animal and human studies have offered some promising, albeit preliminary, evidence that impairments associated with in utero exposure to alcohol can be responsive to intervention. A number of animal studies, for example, have documented some positive effects of neonatal handling, postnatal environment enrichment, and rehabilitative training on rats and mice with perinatal alcohol exposure (see Hannigan et al. 2007; also see article by Idrus and Thomas in this issue, pp. 76–85). Similarly, some notable progress has been made in the last few years in the development of research-based treatments for people with FASD (for reviews, see Chandrasena et al. 2009; Paley and O’Connor 2009; Peadon et al. 2009). Given the early stages of this research, it is not surprising that a number of these studies have methodological limitations. Limited sample size has likely resulted in insufficient power to detect significant differences between treatment and control conditions in some studies. Furthermore, because of their small sample sizes, such studies also have been unable to shed much light on whether various factors, such as IQ, degree of prenatal alcohol exposure, an FAS diagnosis versus other conditions on the FASD continuum, or placement status may moderate treatment outcomes. Moreover, relatively few studies have included follow-up assessments, so it remains unclear whether some of the treatment gains that have been demonstrated immediately following intervention are maintained over time (for some exceptions, see Coles et al. 2009; O’Connor et al. 2006*b*). Nonetheless, these studies highlight the growing momentum in the field to address both the primary deficits and secondary disabilities commonly experienced by people with FASD.

### Parent-Focused Intervention

Because of their significant behavioral, emotional, and cognitive difficulties, children with FASD can be extremely challenging to parent. Indeed, recent studies (Paley et al. 2005, 2006) have highlighted the high levels of parenting stress reported by caregivers of children with FASD. Strains in the parent–child relationship may be evident early on and may increase the risk for negative developmental trajectories. For example, in an early study of a primarily middle-class population (O’Connor et al.1992), women who drank more heavily during pregnancy had infants who displayed higher levels of negative affect in mother–child transactions, compared with infants with less prenatal exposure. In addition, the mothers of these negative-affect infants interacted in ways that were less responsive and developmentally stimulating to their babies, and their infants displayed higher levels of insecure attachment behaviors. In a separate, higher-risk sample of preschool-aged children (O’Connor and Paley 2006), those with moderate or heavy prenatal alcohol exposure exhibited more negative affect when interacting with their mothers than did children with no or only light exposure. The mothers of children exhibiting more negative affect were less emotionally connected to their offspring, and mothers who were less connected to their children had children who experienced higher levels of depressive symptoms. Numerous studies also have documented the high rates of disruptive behaviors, including impulsivity, inattention, hyperactivity, and conduct problems in children with FASD (for a review, see O’Connor and Paley 2009), and such behaviors are likely to tax the internal resources and coping abilities of their caregivers. Such findings underscore the need for interventions that equip caregivers with effective parenting strategies, reduce parenting stress, increase parental self-efficacy, and foster more positive parent–child relationships.

#### Behavioral Consultation

Olson and colleagues (see Bertrand 2009) have developed and evaluated a sustained model of supportive behavioral consultation, Families Moving Forward (FMF), to address the needs of families raising children with FASD. This intervention is designed to increase parental self-efficacy and reduce child behavior problems by providing guidance and instruction to parents and caregivers in the use of strategies that seek to change the environment to reduce problem behavior triggers. Importantly, FMF also seeks to modify some of the cognitions and attitudes that may underlie negative parenting responses to child behavioral problems by recasting such problems as reflective of the neuroteratogenic effects of prenatal alcohol exposure rather than as intentional noncompliance. In addition to the direct support and coaching provided to families, FMF also offers consultation to school personnel and connects families with appropriate community services as needed.

Researchers evaluated the intervention among 52 5- to 11-year-olds with FASD and their caregivers. All participating children exhibited significant impairments in neuropsychological and adaptive functioning, and the study sample included children with FASD who were experiencing significant behavior problems. Families received either the FMF intervention or the community standard of care. Caregivers in the FMF group received 16 in-home sessions every other week over the course of 9 to 11 months. Following the intervention, caregivers participating in the FMF group showed significant improvements in their sense of parenting efficacy, engaged in more self-care behaviors, and were more likely to perceive that their family needs were met, compared with caregivers in the community standard-of-care group. Furthermore, caregivers in the FMF group reported significantly greater improvements in child behavior problems postintervention than did caregivers in the comparison group.

The FMF program is notable for its intensive, sustained approach and focus on the larger family system, as well as broader systems of care. Although some families of children with FASD might not require this level of intervention, there are undoubtedly many families who would benefit from having ongoing guidance in how to parent these children, particularly as their children navigate their way through increasingly complex developmental challenges. Moreover, although the FMF program is described in a written manual with session-by-session instructions for therapists to follow (i.e., it is a manualized intervention), it is also flexible enough that it can be customized to meet the needs of individual families. This initial study validating the efficacy of FMF has laid the groundwork for efforts now under way to disseminate this approach into community settings.

### Educational and Cognitive Interventions

Recent studies have examined the efficacy of various approaches to addressing some of the cognitive, executive functioning, and behavioral impairments that interfere with learning and academic performance among children with FASD. Such children show deficits in verbal and spatial learning, planning, working memory, cognitive flexibility, inhibition, and problem solving, as well as impairments in reading, spelling, and math skills (Duquette and Stodel 2005; Green et al. 2009; Kodituwakku 2009; Mattson et al. 1998; Willoughby et al. 2008). High rates of learning disabilities (Burd et al. 2003) and problematic classroom behaviors (Olson et al. 1992, 1997) also have been observed in this population. Given these challenges, it is not surprising that students with FASD have high rates of disrupted school experiences (e.g., dropping out, suspensions, and expulsions) (Streissguth et al. 2004). Both clinicians and researchers have commented on the importance of modifying both teaching strategies and classroom environments in order to provide increased structure and support for children with FASD (Green 2007; Kalberg and Buckley 2007). Kalberg and Buckley (2007) have noted that when developing and implementing interventions for these children, “It is helpful to think of the environment as an external nervous system of the child, a place where external (environmental) supports can be implemented to bolster the deficit areas of the child” (p. 282). In addition to environmental adaptations that may be implemented in order to accommodate some of the cognitive and behavioral impairments of these individuals, a small but growing number of interventions have focused on enhancing either general learning skills or specific cognitive or academic skill sets.

#### Cognitive Control Therapy

Adnams and colleagues (see Riley et al. 2003) piloted a classroom intervention for 10 students with FAS in the Western Cape Region of South Africa, where rates of FASD are extremely high (May et al. 2009). The students were selected from a group of 64 children identified to have FAS through an active case ascertainment methodology used to screen all first-grade students in the community’s primary schools. The intervention consisted of cognitive control therapy (CCT), which instructs children in strategies that facilitate their ability to acquire and organize information more effectively. Study staff worked collaboratively with regional educational professionals and school personnel in developing the intervention, and children’s cognitive processing deficits and strengths were identified in order to aid in intervention planning.

The children were randomly assigned to either the intervention condition in one classroom in one school or to the control condition in another classroom in another school and were matched on age, grade, first language, socioeconomic statues, and locality of school. Children in the intervention condition received 1 hour of CCT per week from two trained, experienced therapists over the course of 10 months during the school term. The intervention entailed remediation of five metacognitive control domains, by increasing children’s awareness of and effective use of key cognitive strategies, including (1) body position and movements and self awareness, (2) focal attention (scanning and prioritizing information), (3) processing information in the presence of distraction stimuli, (4) controlling external information, and (5) categorizing information.

Compared with the control group, children who received CCT demonstrated marked improvements in classroom behavior. The intervention group also showed qualitative improvements in academic achievement, writing, and communication skills, according to teacher report; improvements in self-efficacy, motivation, self-confidence, and emotionality, according to therapist report; and general school achievement, attitude towards learning, and self-confidence, as reported by school staff. However, the CCT and the control group did not differ significantly from one another on cognitive control and neuropsychological measures. Despite the limitations of the study described by the authors, including a small sample size and a shorter course of CCT than is typical, these initial findings are encouraging. Moreover, as the authors further note, future studies that implement CCT with younger children may be able to capitalize on greater brain adaptability and yield even stronger findings. Additionally, efforts to involve parents or other caregivers in the intervention by teaching them skills to help their children use cognitive control strategies in other settings also might enhance treatment outcomes.

#### Language and Literacy Training

In another study conducted in South Africa, Adnams and colleagues (2007) reported on the efficacy of a school-based language and literacy training (LLT) intervention for 9-year-old children with FASD. LLT focuses on enhancing children’s phonological awareness and promoting the acquisition of pre- and early literacy skills, such as letter knowledge, the ability to manipulate syllables and phonemes, reading real words and nonwords, and semantic skills.

In this study, 40 children with FASD were randomly assigned to either the intervention condition or an FASD control group. The study also included a control group of 25 children who were not exposed to alcohol. Children received two half-hour sessions of LLT twice weekly from an experienced speech and language therapist over the course of 9 months. Sessions were delivered in a group format in a school classroom, and children of similar abilities were assigned to the same group.

Compared with the FASD control group, the LLT group showed significant improvements after treatment in the domains of letter knowledge, syllable manipulation, word and nonword reading, and nonword spelling. However, after the treatment, the LTT group did not differ significantly from the FASD control group on measures of scholastic ability. Moreover, both FASD groups continued to lag significantly behind the nonexposed control group on scholastic measures. Such findings suggest that although it may be possible to enhance specific language and literary skill sets in children with FASD, these improvements may not translate into broader gains in academic achievement. Additionally, these findings may highlight the difficulty of intervening with children in high-risk environments who are likely dealing with additional challenges beyond prenatal exposure (e.g., poverty, ongoing parent alcohol abuse, etc.). Future trials of LLT with this population might examine whether longer-term interventions and/or more individualized instruction would yield greater improvements in measures of scholastic ability.

#### Self-Regulation Intervention

Using a different cognitive-based intervention, Chasnoff and colleagues (see Bertrand 2009) adapted an existing neurocognitive training program, the *Alert Program*^©^ (Williams and Shellenberger 1996) for school-aged children with FASD. This program was adapted to enhance self-regulation skills and remediate executive functioning deficits in 6- to 11-year-old children who had been diagnosed with FAS or ARND and who had been adopted or were in foster care. A total of 78 children were randomly assigned to either the intervention condition or to a control condition. The control group received a comprehensive evaluation and was referred to the community for services such as speech and language therapy, occupational therapy, and physical therapy. Children in the treatment condition also received a complete evaluation for treatment planning and participated in 12 weekly 75-minute neurocognitive rehabilitation group-therapy sessions, whereas their parents participated in a parent education group. Results revealed a significant treatment effect on a parent report measure of executive functioning (see Bertrand 2009). Additional study of this intervention, including obtaining teacher and follow-up data, would help clarify whether these effects generalize to other settings (e.g., school) and are maintained over time.

#### Mathematics Training

Focusing more specifically on a learning disability often noted in children affected by prenatal alcohol exposure, Kable and colleagues (2007) developed a sociocognitive mathematics program for children with FASD. Prior to assignment to treatment and control groups, all caregivers participated in two workshops in which they received education about FASD and instruction in how to promote positive behavioral regulation skills in their children. Participants were then randomly assigned to either a math intervention group or a control group. Both the treatment and the control groups received a neurodevelopmental assessment, and caregivers received guidance in obtaining an appropriate educational placement and in developing an individualized education plan. Children in the math intervention group also received short-term (6 sessions) individualized instruction, and their caregivers received training in how to promote their child’s acquisition of mathematical skills at home and weekly homework to augment the individualized math instruction.

A total of 56 children ages 3–10 with a diagnosis of FAS or partial FAS participated in the program. Parents in both groups reported high levels of satisfaction with the workshops and showed significant improvements in their knowledge of FASD, advocacy topics, and behavioral regulation. Findings revealed that children who received the math intervention in addition to educational support demonstrated greater gains on mathematics outcome measures compared with those who received educational support only (Kable et al. 2007). In one of the few studies to examine the maintenance of treatment effects over time, Coles and colleagues (2009) found that children in the treatment group continued to show these gains at 6-month followup.

#### Working-Memory Strategies

To improve working memory among children with FASD, Loomes and colleagues (2008) developed an intervention to promote the use of rehearsal strategies. Children ages 4–11 were recruited from FASD clinics, various FASD community agencies, and schools. A total of 33 children were randomly assigned to a treatment or control group. Children in the treatment group were instructed to use a simple rehearsal strategy by whispering the items they would be asked to recall.

Children in the experimental condition demonstrated significant improvement in their scores on recalling a series of numbers (i.e., a digit span task) across three sessions (pretest; posttest 1 [immediately after training]; and posttest 2 [6 to 21 days after training]). In contrast, the control group showed no significant change in their scores across the three sessions. Moreover, although the treatment and control groups showed no differences in recall either prior to or immediately following instruction, the treatment group demonstrated significantly greater recall on the digit span task than the control group at the second posttest. Furthermore, the treatment group exhibited behavioral evidence of increased use of rehearsal strategies (e.g., whispering, moving their lips) at posttests 1 and 2, whereas the control group showed no significant change in the use of such strategies across sessions. Similar to many treatment studies with this population, this study included a relatively small sample and did not follow the children over time. Future research in this area would help clarify whether children with FASD are able to maintain the use of a rehearsal strategy over a longer period of time and in other settings, such as in a classroom, where the demand for working memory is likely to be high.

Educational and cognitively based interventions for FASD have yielded some limited but encouraging results, demonstrating that although the negative effects of prenatal alcohol exposure can be pervasive and severe, it seems possible to mitigate some of these impairments with treatment. As most of these studies did not include short-term or long-term followup (for exception, see Coles et al. 2009; Loomes et al. 2008), it remains unclear whether many of the gains demonstrated are maintained over time. Moreover, future studies with larger sample sizes that examine whether certain factors moderate treatment response (e.g., IQ, profile of specific cognitive deficits, level of prenatal alcohol exposure, etc.) may allow for these interventions to be customized in ways that enhance their efficacy with particular subgroups. Additionally, some of these approaches may be enhanced by more actively involving parents or caregivers in the child’s treatment. Notably, the math intervention conducted by Kable and colleagues (2007) and Coles and colleagues (2009), which yielded positive effects at both posttreatment and at the 6-month followup, included coaching parents and caregivers in how to promote their child’s skill acquisition at home. Having consistent and motivated caregivers and professionals who can advocate effectively for these children seems likely to contribute to more positive long-term outcomes. For example, in a study of high-school students with FASD, Duquette and colleagues (2006) proposed that persistence on the part of parents and caregivers played an important role in the social and academic outcomes of these students.

### Adaptive Skills Training

People with FASD show deficits across multiple domains of adaptive functioning, including communication, socialization, and personal and community skills, and such impairments often are more severe than what would be predicted by their cognitive abilities (Jirikowic et al. 2008; Whaley et al. 2001). In a recent study, 5- to 8-year-old children with FASD were compared with a group of typically developing children on a parent-completed scale of independent behavior (Jirikowic et al. 2008). Almost half of the children with FASD required the most intensive levels of support from caregivers, whereas none of the children in the typically developing group required similar levels of support. The demand for such high levels of supervision likely places considerable stress on parents and caregivers. Adaptive functioning deficits limit the child’s opportunities to participate in many of the developmental rites of passage that their typically developing peers are able to experience, and also escalate their risk for adverse outcomes.

#### Social Skills Interventions

Children with FASD demonstrate marked deficits in their social functioning, including difficulties understanding social cues, processing social information, and communicating in social contexts (McGee et al. 2008, 2009; Olson et al. 1998; Streissguth and O’Malley 2000). Such impairments represent an important target for intervention for this population, because poor peer relationships are predictive of early withdrawal from school and delinquency, as well as development of anxious and depressive symptoms (Paetsch and Bertrand 1997; Patterson, et al. 1998; Waldrip et al. 2008).

O’Connor and colleagues (2006*b*) adapted an evidence-based, manualized, parent-assisted social skills intervention, Children’s Friendship Training (CFT) (Frankel and Myatt 2003), for use with children with FASD. CFT is a group-based intervention that teaches social skills to help children be accepted by others rather than rejected, and includes instruction in parent-assisted peer network formation and informational exchange with peers leading to common-ground activities, peer entry, and play skills. Children learn these skills through didactic instruction on the basic rules of social behavior, modeling, rehearsal, and coached practice with performance feedback during treatment sessions. To promote maintenance and generalization of the newly learned skills, in-session instruction is augmented with parent-assisted activities outside of the sessions, including rehearsal of skills at home, homework assignments (e.g., having a play date), and coaching by parents during interactions with peers.

In a randomized controlled efficacy study, 100 children with a history of moderate to heavy prenatal alcohol exposure were recruited from outpatient hospital clinics, community health care providers, and schools. To be eligible for the study, participants had to be between 6 and 12 years of age, have a verbal IQ of at least 70 so that they would be able to comprehend the didactic component of the intervention, and have one parent or care-giver who could consistently attend the training. All children who qualified for the study had a diagnosis of FAS, partial FAS, or ARND. Children were randomly assigned to the experimental group (CFT) or to a delayed treatment condition (DTC). A total of 96 children and their parents completed the baseline and posttreatment phases of the study. Children participated in weekly 90-minute group sessions over the course of 12 weeks, while their parents participated in concurrent groups, receiving instruction on how to promote the use of the child’s new social skills in other environments (e.g., before and after school, or at the park).

Compared with children in the DTC group, those who received CFT showed significantly greater improvement in their knowledge of appropriate social behavior and were rated by their parents as having better social skills and fewer behavior problems after treatment on the Social Skills Rating System (SSRS) (Gresham and Elliott 1990), a standardized parent-report measure. Moreover, these treatment gains were maintained at a 3-month follow-up assessment. To examine the clinical significance of these findings, parent ratings of children’s social skills and behavior problems in the CFT group were compared with the standardized normative sample for the SSRS. Results indicated that at both posttreatment and the 3-month followup, mean scores for the CFT group moved from the clinical range to within the normative range but still remained significantly different from the normative sample mean. Given the significant neurocognitive impairments that characterize many children with FASD, it is not surprising that there may be some limits to the treatment gains that can be yielded by a psychosocial intervention. However, although it may not be possible to completely normalize the social behavior of these children, the finding that their social functioning had moved to the normative range post-treatment and remained there at followup suggests that this type of intervention can have a significant clinical impact.

As a followup to this study, CFT was subsequently disseminated into a community mental health center and implemented with groups that included children both with and without FASD (O’Connor et al. 2009). CFT was compared with the clinic’s standard of care (SOC) social skills training, which was process oriented and behaviorally based and involved group discussion and cooperative projects but did not include a parent component. The CFT parent groups were conducted in both English and Spanish to accommodate the high percentage of Spanish-speaking parents in this community setting. A total of 85 families were enrolled in the study, and 67 families completed treatment. Completers and noncompleters did not differ on either important demographic characteristics or prenatal exposure. Preliminary analyses indicated that children in the CFT group demonstrated significantly greater gains in their knowledge of appropriate social behavior than children in the SOC group. Similarly, parents of children in the CFT group rated their children as showing greater improvement in social skills than parents of children in the SOC group. Parent satisfaction was higher in the CFT group than in the SOC group, with 92 percent of parents in the CFT condition endorsing the belief that the intervention had improved their children’s ability to get along with peers, whereas only 60 percent in the SOC condition endorsed this belief. Therapist satisfaction with CFT also was high, with 92 percent of the therapists who were trained to deliver CFT stating that they would like to see the intervention permanently adopted in their center. Findings from this effectiveness study are encouraging as they demonstrate that a research-based intervention to address social impairments in children with FASD could be successfully translated into a community setting and that these children can be treated in groups that include children with other kinds of behavioral, emotional, and social difficulties.

#### Safety Skills

Parents of children with FASD frequently report concerns about their children’s lack of safety awareness. In general, children with disabilities and behavioral problems are at an elevated risk for unintentional injuries (Sherrard et al. 2004), and given their impulsivity, lack of behavioral inhibition, poor judgment, and difficulty generalizing skills across contexts, children with FASD likely fall into this high-risk group as well. With this in mind, Coles and colleagues (2007) designed a computer-based intervention designed to improve fire- and street-safety skills in children with FASD (Coles et al. 2007)]. The authors noted that the computer-game format was selected because most children find these games engaging; it allows them to learn at their own rate with repetition of material if needed; and the format presents an opportunity to practice skills in a “virtual world” that provides visual, auditory, and motor input, as well as performance feedback.

Thirty-two children ages 4 to 10 with diagnoses of FAS or partial FAS were recruited for the study. Children were divided into one of two groups, and each group was taught appropriate rules and behavioral sequences in response to either one of two situations: a fire in their home, or crossing a city street. Children were guided through the computerized training by an animated figure that presented information on fire safety or street safety in small, incremental steps. Children were reinforced for correct actions and received feedback if they engaged in dangerous actions and then were taken back to the beginning of the behavioral sequence until they responded correctly. For more complex behaviors, children were not allowed to perform the sequence incorrectly, to prevent them from learning unsafe behaviors that might be more challenging to extinguish.

Children’s knowledge and skills were assessed pretest, posttest, and during a 1-week followup, and each group served as the control group for the other. Children in each intervention group (fire safety or street safety) demonstrated significant gains from pretest to posttest and from pretest to followup in safety-related knowledge and appropriate behavioral responses and significantly greater gains in comparison to the control group. Children in the treatment groups also demonstrated generalization of their knowledge and skills in a “real-world” situation at both posttest and followup. Findings from this study suggest that it may be possible to apply computer-based interventions to the teaching of other safety-related skills, such as water safety or stranger awareness, to children with FASD.

## Conclusion and Future Directions

The studies described in this review attest to the notable progress that has been made in recent years in developing and testing interventions designed to remediate impairments in multiple domains of functioning among people with FASD. Such studies offer some promise for families affected by FASD who often have considerable difficulty accessing appropriate services. Indeed, the results yielded by these studies begin to lay the foundation for a number of potentially fruitful avenues of investigation that will further expand the capacity of both clinicians and researchers to respond to the treatment needs of this vastly underserved population.

The notion of a continuum of disorders related to in utero exposure to alcohol highlights that there is considerable variability in how FASD is manifested across different individuals, and, similarly, individual responses to interventions differ as well. Future studies that include larger sample sizes may help identify how various individual, family, and systems factors may moderate treatment response. Such factors might include where individuals fall on the FASD continuum; their particular profile of neurocognitive deficits; and the presence or absence of comorbid psychiatric conditions, parental involvement or commitment, social support, or availability of community resources. Elucidating how these factors might play a role in treatment outcomes will allow researchers and clinicians to refine interventions so that they may better serve affected individuals and their families.

Many of the studies reviewed here, although demonstrating some positive posttreatment effects, did not include follow-up assessments. There would be considerable value in examining whether individuals with FASD continue to benefit from intervention after they are no longer receiving it and whether they continue to demonstrate treatment gains in less predictable environments than highly controlled research settings. Such followup is particularly important with this population, given their difficulties with learning and retaining information. Moreover, because people with FASD often must also contend with a range of postnatal risk factors, such as chaotic caregiving environments or unstable placements, it may be challenging for them to retain and apply newly learned skills or knowledge under highly stressful circumstances. The few studies that have included followup assessments (e.g., Coles et al. 2009; O’Connor et al. 2006*b*) suggest that treatment for people with FASD can yield sustainable gains, but it would be important to examine the longer-term outcomes of other interventions and what factors might promote or hinder the maintenance of treatment gains.

Most research-based interventions for this population have focused on school-aged children, and thus there is a need to develop treatments for younger children, as well as adolescents and adults. Treatments for alcohol-exposed infants and toddlers have the potential to capitalize on early neuroplasticity, and mitigating some of the early manifestations of FASD (e.g., poor self-regulation, heightened response to stress) may help pave the way for more positive developmental trajectories for these children. Interventions for people with FASD transitioning into adulthood are critical because substance use and abuse problems, high-risk sexual behavior, and illegal activities may emerge or worsen during this developmental period. Parents and caregivers may find it challenging to provide adequate monitoring in order to keep their adolescent and even adult children, as well as the surrounding community, safe. Treatment approaches aimed at decreasing participation in high-risk activities might focus on enhancing skills in decision-making, problem solving, and behavioral regulation in adolescents and adults with FASD, as well as better equipping parents to supervise their children’s whereabouts, activities, and peer associations.

Finally, it is imperative that interventions with established efficacy are translated and evaluated in community settings in order to increase the accessibility of services for this population. Previous research suggests that some practitioners have believed that having a diagnosis would not improve outcomes for affected individuals (Alaska FAS Prevention Steering Committee, 1995). If health care providers are aware that there are effective treatment options available for individuals with FASD, they may be more vigilant in identifying signs of these disorders in the first place and may better appreciate the potential benefit in referring families for appropriate services. Taken together, these suggestions for developing, evaluating, and disseminating a broad range of treatment approaches are consistent with the recommendations of the National Task Force on Fetal Alcohol Syndrome and Fetal Alcohol Effect (Olson et al. 2009), the Five Year Strategic Research Plan on FASD from the National Institute on Alcohol Abuse and Alcoholism (NIAAA 2006), and various research initiatives sponsored by NIAAA, the Centers for Disease Control and Prevention, and the Substance Abuse and Mental Health Services Administration (SAMHSA) Fetal Alcohol Spectrum Disorders Center for Excellence. Building collaborative systems of care for this population is a critical step in decreasing the societal burden of FASD and its personal costs to families, and increasing the likelihood that affected individuals will be able to lead productive and fulfilling lives.

## References

[b1-arh-34-1-64] Adnams CM, Kodituwakku P, Hay A (2001). Patterns of cognitive-motor development in children with Fetal Alcohol Syndrome from a community in South Africa. Alcoholism: Clinical and Experimental Research.

[b2-arh-34-1-64] Adnams CM, Sorour P, Kalberg WO (2007). Language and literacy outcomes from a pilot intervention study for children with Fetal Alcohol Spectrum Disorders in South Africa. Alcohol.

[b3-arh-34-1-64] Alaska Fetal Alcohol Syndrome Prevention Steering Committee (1995). Alcohol related knowledge, attitude, belief and behavior (KABB) surveys of Alaskan health professionals. Alaska Medicine.

[b4-arh-34-1-64] Alati R, Clavarino A, Najman JM (2008). The developmental origin of adolescent alcohol use: Findings from the Mater University Study of Pregnancy and its outcomes. Drug and Alcohol Dependency.

[b5-arh-34-1-64] Áragon AS, Coriale G, Fiorentino D (2008). Neuropsychological characteristics of Italian children with Fetal Alcohol Spectrum Disorders. Alcoholism: Clinical and Experimental Research.

[b6-arh-34-1-64] Astley SJ, Stachowiak J, Clarren SK, Clausen C (2002). Application of the fetal alcohol syndrome facial photographic screening tool in a foster care population. Journal of Pediatrics.

[b7-arh-34-1-64] Bertrand J (2009). Interventions for children with fetal alcohol spectrum disorders: Overview of findings for five innovative research projects. Research in Developmental Disabilities.

[b8-arh-34-1-64] Bonthius DJ, West JR (1990). Alcohol-induced neuronal loss in developing rats: increased brain damage with binge exposure. Alcoholism: Clinical and Experimental Research.

[b9-arh-34-1-64] Burd L (2007). Interventions in FASD: We must do better. Child: Care, Health, and Development.

[b10-arh-34-1-64] Burd L, Carlson C, Kerbeshian J (2007). Fetal Alcohol Spectrum Disorders and Mental Illness. International Journal on Disability and Human Development.

[b11-arh-34-1-64] Burd L, Klug MG, Martsolf JT, Kerbeshian J (2003). Fetal alcohol syndrome: neuropsychiatric phenomics. Neurotoxicology and Teratology.

[b12-arh-34-1-64] Burd L, Selfridge R, Klug M, Bakko S (2004). Fetal alcohol syndrome in the United States corrections system. Addiction Biology.

[b13-arh-34-1-64] Chandrasena AN, Mukherjee RAS, Turk J (2009). Fetal alcohol spectrum disorders: An overview of interventions for affected individuals. Child and Adolescent Mental Health.

[b14-arh-34-1-64] Coggins TE, Timler GR, Olswang LB (2007). A state of double jeopardy: Impact of prenatal alcohol exposure and adverse environments on the social communicative abilities of school-age children with Fetal Alcohol Spectrum Disorders. Language, Speech, and Hearing Services in Schools.

[b15-arh-34-1-64] Coles CD, Kable JA, Taddeo E (2009). Math performance and behavior problems in children affected by prenatal alcohol exposure: Intervention and follow-up. Journal of Developmental and Behavioral Pediatrics.

[b16-arh-34-1-64] Coles CD, Strickland DC, Padgett L, Bellmoff L (2007). Games that “work”: Using computer games to teach alcohol-affected children about fire and street safety. Research in Developmental Disabilities.

[b17-arh-34-1-64] Day NL, Richardson GA (2004). An analysis of the effects of prenatal alcohol exposure on growth: A teratologic model. American Journal of Medical Genetics.

[b18-arh-34-1-64] Downing C, Balderrama-Durbin C, Broncucia H Ethanol teratogenesis in five inbred strains of mice. Alcoholism: Clinical and Experimental Research.

[b19-arh-34-1-64] Duquette C, Stodel DL (2005). School experiences of students with Fetal Alcohol Spectrum Disorder. Exceptionality Education Canada.

[b20-arh-34-1-64] Duquette C, Stodel DL, Fullarton S, Hagglund K (2006). Persistence of high school experiences of adolescents and young adults with Fetal Alcohol Spectrum Disorder. Journal of Intellectual and Developmental Disabilities.

[b21-arh-34-1-64] Elliot EJ, Payne J, Haan E, Bower C (2006). Diagnosis of foetal alcohol syndrome and alcohol use in pregnancy: A survey of paediatricians’ knowledge, attitudes, and practice. Journal of Pediatrics and Child Health.

[b22-arh-34-1-64] Elliot EJ, Payne J, Haan E (2008). Fetal Alcohol Syndrome: A prospective national surveillance study. Archives of Disease in Childhood.

[b23-arh-34-1-64] FASD Regional Training Centers Consortium (2007). Educating health professionals about fetal alcohol spectrum disorders. American Journal of Health Education.

[b24-arh-34-1-64] Frankel F, Myatt R (2003). Children’s Friendship Training.

[b25-arh-34-1-64] Gahagan S, Sharpe TT, Brimacombe MP (2006). Pediatricians’ knowledge, training, and experience in the care of children with fetal alcohol syndrome. Pediatrics.

[b26-arh-34-1-64] Green JH (2007). Fetal Alcohol Spectrum Disorders: Understanding the effects of prenatal alcohol exposure and supporting students. Journal of School Health.

[b27-arh-34-1-64] Green CR, Mihic AM, Nikkel SM (2009). Executive function deficits in children with fetal alcohol spectrum disorders (FASD) measured using the Cambridge Neuropsychological Tests Automated Battery (CANTAB). Journal of Child Psychology and Psychiatry.

[b28-arh-34-1-64] Gresham FM, Elliott S (1990). The Social Skills Rating System.

[b29-arh-34-1-64] Guerri C, Bazinet A, Riley EP (2009). Foetal Alcohol Spectrum Disorders and alterations in brain and behavior. Alcohol and Alcohol Research.

[b30-arh-34-1-64] Hannigan JH, O’leary-Moore SK, Berman RF (2007). Postnatal environmental or experiential amelioration of neurobehavioral effects of perinatal alcohol exposure in rats. Neuroscience and Biobehavioral Review.

[b31-arh-34-1-64] Jacobson SW, Carr LG, Croxford J (2006). Protective effects of the alcohol dehydrogenase-ADH1B allele in children exposed to alcohol during pregnancy. Journal of Pediatrics.

[b32-arh-34-1-64] Jirikowic T, Kartin D, Olson HC (2008). Children with Fetal Alcohol Spectrum Disorders: A descriptive profile of adaptive function. Canadian Journal of Occupational Therapy.

[b33-arh-34-1-64] Jones KL (2006). The role of genetic susceptibility for maternal alcohol metabolism in determining pregnancy outcome. Journal of Pediatrics.

[b34-arh-34-1-64] Jones KL, Smith DW (1973). Recognition of the fetal alcohol syndrome in early infancy. Lancet.

[b35-arh-34-1-64] Jones KL, Smith DW, Ulleland CN, Streissguth AP (1973). Pattern of malformations in offspring of alcoholic mothers. Lancet.

[b36-arh-34-1-64] Kable JA, Coles CD, Taddeo E (2007). Sociocognitive habilitation using the Math Interactive Learning Experience program for alcohol affected children. Alcoholism:. Clinical and Experimental Research.

[b37-arh-34-1-64] Kalberg WO, Buckley D (2007). FASD: What types of intervention and rehabilitation are useful?. Neuroscience and Biobehavioral Reviews.

[b38-arh-34-1-64] Kalberg WO, Provost B, Tollison SJ (2006). Comparison of motor delays in young children with fetal alcohol syndrome to those with prenatal alcohol exposure and with no prenatal alcohol exposure. Alcohol Clinical Exposure Research.

[b39-arh-34-1-64] Kodituwakku PW (2007). Defining the behavioral phenotype in children with fetal alcohol spectrum disorders: A review. Neuroscience and Biobehavioral Reviews.

[b40-arh-34-1-64] Kodituwaku PW (2009). Neurocognitive profile in children with fetal alcohol spectrum disorders. Developmental Disabilities Research Reviews.

[b41-arh-34-1-64] Kodituwaku PW, Adnams CM, Hay A (2006a). Letter and category fluency in children with Fetal Alcohol Syndrome from a community in South Africa. Journal of Studies on Alcohol.

[b42-arh-34-1-64] Kodituwakku PW, Coriale G, Fiorentino D (2006b). Neurobehavioral characteristics of children with Fetal Alcohol Spectrum Disorders in Communities from Italy: Preliminary results. Alcoholism: Clinical and Experimental Research.

[b43-arh-34-1-64] Loomes C, Rasmussen C, Pei J (2008). The effects of rehearsal training on working memory span of children with fetal alcohol spectrum disorder. Research in Developmental Disabilities.

[b44-arh-34-1-64] Lupton C, Burd L, Harwood R (2004). Cost of fetal alcohol spectrum disorders. American Journal of Medical Genetics.

[b45-arh-34-1-64] Mattson SN, Riley EP, Gramling L (1998). Neuropsychological comparison of alcohol-exposed children with or without physical features of fetal alcohol syndrome. Neuropsychology.

[b46-arh-34-1-64] May PA, Gossage JP, Kalberg WO (2009). Prevalence and epidemiologic characteristics of FASD from various research methods with an emphasis on recent in-school studies. Developmental Disabilities Research Review.

[b47-arh-34-1-64] May PA, Gossage JP, Marais AS (2007). The epidemiology of fetal alcohol syndrome and partial FAS in a South African community. Drug and Alcohol Dependence.

[b48-arh-34-1-64] May PA, Gossage JP, Marais AS (2008). Maternal risk factors for fetal alcohol syndrome and partial FAS in a South Africa: A third study. Alcoholism: Clinical and Experimental Research.

[b49-arh-34-1-64] Mcgee CL, Bjorkquist OA, Price JM (2009). Social information processing in children with histories of heavy prenatal alcohol exposure. Journal of Abnormal Child Psychology.

[b50-arh-34-1-64] Mcgee CL, Fryer SL, Bjorkquist OA (2008). Social problem solving deficits in adolescents with prenatal exposure to alcohol. American Journal of Drug and Alcohol Abuse.

[b51-arh-34-1-64] Mekonnen R, Noonan K, Rubin D (8924). Achieving better health care outcomes for children in foster care. Pediatric Clinics of North America.

[b52-arh-34-1-64] National Institute on Alcohol Abuse and Alcoholism Five year strategic plan (FY 07-11): Alcohol across the lifespan.

[b53-arh-34-1-64] O’Connor MJ, Frankel F, Paley B (2006b). A controlled social skills training for children with fetal alcohol spectrum disorders. Journal of Consulting and Clinical Psychology.

[b54-arh-34-1-64] O’Connor MJ, Mccracken J, Best A (2006a). Under recognition of prenatal alcohol exposure in a child inpatient psychiatric setting. Mental Health Aspects of Developmental Disabilities.

[b55-arh-34-1-64] O’Connor MJ, Paley B (2006). The relationship of prenatal alcohol exposure and the postnatal environment to child depressive symptoms. Journal of Pediatric Psychology.

[b56-arh-34-1-64] O’Connor MJ, Paley B (2009). Psychiatric conditions associated with prenatal alcohol exposure. Developmental Disabilities Research Review.

[b57-arh-34-1-64] O’Connor MJ, Paley B, Laugeson E (2009). Lessening the risk of social skills deficits in children exposed to alcohol prenatally: An evidence based intervention in a community setting.

[b58-arh-34-1-64] O’Connor MJ, Shah B, Whaley S (2002). Psychiatric illness in a clinical sample of children with prenatal alcohol exposure. American Journal of Drug and Alcohol Abuse.

[b59-arh-34-1-64] O’Connor MJ, Sigman M, Kasari C (1992). Attachment behavior of infants exposed prenatally to alcohol: Mediating effects of infant affect and mother-infant interaction. Development and Psychopathology.

[b60-arh-34-1-64] Olson HC, Feldman JJ, Streissguth AP (1998). Neuropsychological deficits in adolescents with fetal alcohol syndrome: Clinical findings. Alcoholism: Clinical and Experimental Research.

[b61-arh-34-1-64] Olson HC, Jirikowic T, Kartin D, Astley S (2007). Responding to the challenge of early intervention for Fetal Alcohol Spectrum Disorders. Infants and Young Children.

[b62-arh-34-1-64] Olson HC, Ohlemiller MM, O’Connor MJ (2009). National Task Force on Fetal Alcohol Syndrome and Fetal Alcohol Effect A Call to Action: Advancing Essential Services and Research on Fetal Alcohol Spectrum Disorders: A report of the National Task Force on Fetal Alcohol Syndrome and Fetal Alcohol Effect.

[b63-arh-34-1-64] Olson HC, Oti R, Gelo J, Beck S (2009). Family matters: Fetal Alcohol Spectrum Disorders and the family. Developmental Disabilities Research Reviews.

[b64-arh-34-1-64] Olson HC, Sampson PD, Barr H (1992). Prenatal exposure to alcohol and school problems in late childhood: A longitudinal prospective study. Developmental Psychopathology.

[b65-arh-34-1-64] Olson HC, Streissguth A, Sampson PD (1997). Association of prenatal alcohol exposure with behavioral and learning problems in early adolescence. Journal of the American Academy of Child and Adolescent Psychiatry.

[b66-arh-34-1-64] Paetch JJ, Bertrand LD (1997). The relationship between peer, social, and school factors, and delinquency among youth. Journal of School Health.

[b67-arh-34-1-64] Paley B, O’Connor MJ (2007). Neurocognitive and neurobehavioral impairments in individuals with fetal alcohol spectrum disorders: Recognition and assessment. International Journal on Disability and Human Development.

[b68-arh-34-1-64] Paley B, O’Connor MJ (2009). Intervention for individuals with Fetal Alcohol Spectrum Disorders: Treatment approaches and case management. Developmental Disabilities Research Reviews.

[b69-arh-34-1-64] Paley B, O’Connor MJ, Baillie S (2009). Integrating case topics in medical school curriculum to enhance multiple skill learning: Using Fetal Alcohol Spectrum Disorders as an exemplary case. Academic Psychiatry.

[b70-arh-34-1-64] Paley B, O’Connor MJ, Frankel F, Marquardt R (2006). Predictors of stress in parents of children with Fetal Alcohol Spectrum Disorders. Journal of Developmental and Behavioral Pediatrics.

[b71-arh-34-1-64] Paley B, O’Connor MJ, Kogan N, Findlay R (2005). Prenatal alcohol exposure, child externalizing behavior, and maternal stress. Parenting: Science and Practice.

[b72-arh-34-1-64] Patterson GR, Forgatch MS, Yoerger KL, Stoolmiller M (1998). Variables that initiate and maintain and early-onset trajectory for juvenile offending. Development and Psychopathology.

[b73-arh-34-1-64] Peadon E, Rhys-Jones B, Bower C, Elliott EJ (2009). Systematic review of interventions for children with Fetal Alcohol Spectrum Disorders. BMC Pediatrics.

[b74-arh-34-1-64] Premji S, Benzies K, Serrett K, Hayden KA (2006). Research-based interventions for children and youth with a Fetal Alcohol Spectrum Disorder: Revealing the gap. Child: Care, Health, and Development.

[b75-arh-34-1-64] Rasmussen C (2005). Executive functioning and working memory in Fetal Alcohol Spectrum Disorder. Alcoholism: Clinical and Experimental Research.

[b76-arh-34-1-64] Rasmussen C, Bisanz J (2009). Executive functioning in children with Fetal Alcohol Spectrum Disorders: Profiles and age-related differences. Child Neuropsychology.

[b77-arh-34-1-64] Riley EP, Mattson SN, Li T (2003). Neurobehavioral consequences of prenatal alcohol exposure: An international perspective. Alcoholism: Clinical and Experimental Research.

[b78-arh-34-1-64] Riley EP, Mcgee CL Fetal alcohol spectrum disorders: An overview with emphasis on changes in brain and behavior. Experimental Biology and Medicine.

[b79-arh-34-1-64] Roebuck TM, Simmons RW, Mattson SN, Riley EP (1998). Prenatal exposure to alcohol affects the body to maintain postural balance. Alcoholism: Clinical and Experimental Research.

[b80-arh-34-1-64] Sampson PD, Streissguth AP, Bookstein FL (1997). Incidence of fetal alcohol syndrome and prevalence of alcohol-related neurodevelopmental disorder. Teratology.

[b81-arh-34-1-64] Sherrard J, Ozanne-Smith J, Staines C (2004). Prevention of unintentional injury to people with intellectual disability: A review of the evidence. Journal of Intellectual Disability Research.

[b82-arh-34-1-64] Smith DK, Johnson AB, Pears KC (2007). Child maltreatment and foster care: Unpacking the effects of prenatal and postnatal parental substance use. Child Maltreatment.

[b83-arh-34-1-64] Stade B, Ali A, Bennett D The burden of prenatal alcohol exposure to alcohol: Revised measurement of cost. Canadian Journal of Clinical Pharmacology.

[b84-arh-34-1-64] Stratton KR, Howe CJ, Battaglia FC (1996). Fetal Alcohol Syndrome: Diagnosis, Epidemiology, Prevention, and Treatment.

[b85-arh-34-1-64] Streissguth AP (2007). Offspring effects of prenatal alcohol exposure from birth to 25 years: The Seattle Prospective Longitudinal Study. Journal of Clinical Psychology in Medical Settings.

[b86-arh-34-1-64] Streissguth AP, Bookstein FL, Barr HM (2004). Risk factors for adverse life outcomes in Fetal Alcohol Syndrome and Fetal Alcohol Effects. Developmental and Behavioral Pediatrics.

[b87-arh-34-1-64] Streissguth AP, O’Malley K (2000). Neuropsychiatric implications and long-term consequences of Fetal Alcohol Spectrum Disorders. Seminars in Clinical Neuropsychiatry.

[b88-arh-34-1-64] Thanh NX, Jonsson E Costs of Fetal Alcohol Spectrum Disorder in Alberta, Canada. Canadian Journal of Clinical Pharmacology.

[b89-arh-34-1-64] Viljoen DL, Gossage JP, Brooke L (2005). Fetal Alcohol Syndrome epidemiology in a South African community: A second study of a very high prevalence area. Journal of Studies on Alcohol.

[b90-arh-34-1-64] Waldrip AM, Malcolm KT, Jensen-Campbell LA (2008). With a little help from your friends: The importance of high-quality friendships on early adolescent adjustment. Social Development.

[b91-arh-34-1-64] Walthall JC, O’Connor MJ, And Paley B (2008). A comparison of psychopathology in children with and without prenatal alcohol exposure. Mental Health Aspects of Developmental Disabilities.

[b92-arh-34-1-64] Warren K, Floyd L, Calhoun F (2004). Consensus statement on FASD.

[b93-arh-34-1-64] Whaley SE, O’Connor MJ, Gunderson B (2001). Comparison of the adaptive functioning of children prenatally exposed to alcohol to a nonexposed clinical sample. Alcoholism: Clinical and Experimental Research.

[b94-arh-34-1-64] Williams MS, Shellenberger S (1996). How does your engine run? A leader’s guide to the Alert Program^©^ for self-regulation.

[b95-arh-34-1-64] Willoughby KA, Sheard ED, Nash K, Rovet J (2008). Effects of prenatal alcohol exposure on hippocampal volume, verbal learning, and verbal and spatial recall in late childhood. Journal of the International Neuropsychological Society.

